# The influence of the national drug price negotiation policy reform on the medical expenses of patients in Xuzhou City: an interrupted time series analysis

**DOI:** 10.3389/fpubh.2024.1381786

**Published:** 2024-06-05

**Authors:** Zhaohui Qin, Meng He, Huangying Shen, Sha Liu, Shuo Xu, Lijiang Chen

**Affiliations:** ^1^Research Center for Medical and Health Emergency Rescue of The Second Clinical Medical School, Xuzhou Medical University, Xuzhou, Jiangsu, China; ^2^School of Management, Xuzhou Medical University, Xuzhou Medical University, Xuzhou, Jiangsu, China; ^3^School of Medicine and Health Management, Tongji Medical College, Huazhong University of Science and Technology, Wuhan, China; ^4^The First Hospital of Nanping, Nanping, China

**Keywords:** drug cost, national drug price negotiation, health policy, interrupted time series analysis, patient burden

## Abstract

**Background:**

To reduce the burden of patients’ medical care, the Xuzhou Municipal Government has initiated an exploratory study on the supply model and categorized management of nationally negotiated drugs. This study aims to understand the extent to which Xuzhou’s 2021 reform of the National Drug Price Negotiation (NDPN) policy has had a positive impact on the healthcare costs of individuals with different types of health insurance.

**Methods:**

The Interrupted Time Series Analysis method was adopted, and the changes in average medical expenses per patient, average medical insurance payment cost per patient and actual reimbursement ratio were investigated by using the data of single-drug payments in Xuzhou from October 2020 to October 2022.

**Results:**

Following the implementation of the policy, there was a significant decrease in the average medical expenses per patient of national drug negotiation in Xuzhou, with a reduction of 62.42 yuan per month (*p* < 0.001). Additionally, the average medical insurance payment cost per patient decreased by 44.13 yuan per month (*p* = 0.01). Furthermore, the average medical expenses per patient of urban and rural medical insurance participants decreased by 63.45 yuan (*p* < 0.001), and the average monthly medical insurance payment cost per patient decreased by 57.56 yuan (*p* < 0.04). However, the mean total medical expenditures for individuals enrolled in employee medical insurance decreased by 63.41 yuan per month (*p* < 0.001), whereas the monthly decrease was 22.11 yuan per month (*p* = 0.21). On the other hand, there was no discernible change in the actual reimbursement ratio.

**Conclusion:**

After the adoption of the NDPN policy, a noticeable decline has been observed in the average medical expenses per patient and the mean cost of the average medical insurance payment per patient, although to a limited extent. Notably, the reduction in employee medical insurance surpasses that of urban and rural medical insurance.

## Introduction

Within the context of China, medical expenses have emerged as a significant concern, as their escalation has exerted a mounting impact on the overall burden borne by the nation and the *per capita* economic load. Over the past 15 years, China has witnessed a consistent annual growth rate of >9% in its medical expenditure (1). The growth rate of medical expenditure is faster than that of gross domestic product, and the reimbursement of high-priced drugs is limited.

China is not alone in facing the financial burden of rising drug costs (2, 3). The increasing attention paid by many countries to the impact of drug prices on the economic stress of individuals has led to the development of various strategies aimed at reducing drug prices. These approaches encompass internal reference pricing, external reference pricing (4) and special pricing agreements (5). Internal Reference Pricing (IRP) is the practice of setting or negotiating reimbursement of drug prices by reference to domestic prices for identical, similar or therapeutically equivalent drugs. In New Zealand, for example, manufacturers can charge higher prices, but government reimbursement is limited based on the reference price (4). External Reference Pricing (ERP), is a price control mechanism whereby the government takes into account the prices of medicines in other countries in order to inform or set prices in its own country. For example, in Japan, drug prices are compared with the average price of drugs in four reference countries and prices are dynamically adjusted (6). Special Pricing Agreements (SPAs) are innovative agreements between governments and manufacturers for payers and pharmaceutical companies to align on value, speed to market and risk. For example, Australia’s SPA allows manufacturers to set a higher public bid price, which is offset by a rebate to the government based on predetermined criteria (4, 7).

In response to increasing expenditure on medicines, the Chinese government introduced a National Drug Price Negotiation (NDPN) policy in 2016, whereby the state centrally organizes negotiations to determine drug prices. The main objective of the policy is to promote the centralization of drugs in public hospitals and reduce drug costs (8). In the context of this policy, insured persons have access to reimbursement for negotiated medicines, which improves cost-effectiveness, and thereby reduces the burden (9, 10). Since the implementation of the National Drug Policy, drug prices have been reduced by an average of 60.1%, reducing the burden on patients by >$90 billion (11).

Prior to the reform of the national-negotiated drug policy, most drugs were listed in hospitals before entering the health insurance scheme, giving the drugs enough time to go through the process of market promotion, accumulation of experience in clinical use and recognition by clinical experts for widespread use. However, after the reform of the national negotiation drug policy, it has become “enter the medical insurance first, and then enter the hospital,” which has posed a challenge to the access procedure of the national negotiation drugs in the medical institutions and the extensive use of drugs by the clinicians within a short period of time. To address the issue of “difficulty in seeing a doctor,” the Chinese government has proposed to classify and manage the negotiated medicines by taking into account factors such as clinical value and patients’ reasonable needs for medicines. The designated retail pharmacies will be included in the scope of supply of the negotiated drugs, and together with the designated medical institutions, they will be reimbursed for the negotiated drugs through “dual channels.”

Xuzhou City in Jiangsu Province is located on the eastern coast of China, with a total population of more than 9 million people in 10 municipal districts, and a GDP of RMB 845.78 billion in 2022, making it a city with a medium level of economic development in the eastern region of China. In September 2021, Xuzhou Municipality issued the “Notice on Matters Related to the Implementation of the National Health Insurance Negotiated Drugs “Dual-channel” Management Mechanism,” which raised the treatment of individually-paid drugs for Xuzhou Municipal Employees’ Health Insurance from 55 to 70%, and the treatment of individually-paid drugs for Xuzhou Municipal Residents’ Health Insurance from 50 to 60%. Xuzhou City has a total of 175 nationally negotiated drugs in 2023 that are “dual-channel” drugs. In other regions, Sichuan Province in 2023, a total of 154 kinds of drugs to implement single-line payment management, lower than Xuzhou City, 175 kinds. In Hunan Province, the catalog of “dual-channel” single-line payment management medicines in 2023 includes 233 types of medicines higher than Xuzhou City.

There are two main types of social medical insurance systems in China’s social medical insurance sector. The basic medical insurance system for urban and rural residents mainly caters to the healthcare needs of rural residents, urban residents, students and children. In contrast, the basic medical insurance for urban workers is specifically designed to meet the healthcare needs of urban workers (12, 13). In terms of system design, the two types of health insurance show fundamental differences due to different resource endowments, with the actual reimbursement rate and affordability of urban and rural residents lower than those of urban workers (14).

We explore the impact of Xuzhou City’s 2021 reform of the NDPN policy through the changes in average medical expenses per patient, average medical insurance payment cost per patient and actual reimbursement ratio, three indicators at the two levels of urban workers’ and urban and rural residents’ medical insurance participation, respectively. The findings of this study should offer empirical insights and pertinent recommendations to enhance national policies, advance hospital reform and alleviate the financial burden on patients.

## Methods

### Study design

The present study employs the methodology of Interrupted Time Series Analysis (ITSA) to examine the effects of policies on individuals by using purchase data obtained from the Medical Insurance Bureau. The analysis covers the period spanning from October 2020 to October 2022. In September 2021, Xuzhou City increased the separate payment drug entitlement from 55 to 70% for patients attending employee health insurance and from 50 to 60% for patients attending resident health insurance. Therefore, the policy time point of September 2021 has been set for this study.

### Data sources

The aforementioned data are sourced from the medical insurance data information system of the Xuzhou Municipal Medical Insurance Bureau, a government-managed entity that restricts public access to safeguard patient confidentiality. This database encompasses comprehensive medical and insurance information pertaining to all inhabitants within the specified region.

### Statistical analysis

The study employed the ITSA model to examine the effects of modifications in NDPN policy on the hospitalization costs of residents. ITSA is a highly regarded research design that enables the explicit identification of the intervention time point, making it the preferred analytical approach for evaluating the longitudinal impact of interventions (15, 16). This study aimed to examine the involvement of insured patients in the implementation of the NDPN policy as an intervention strategy in Xuzhou City, Jiangsu Province, in September 2021. The independent variable was measured in months, with a pre- and post-intervention period of 10 months (October 2020–October 2021 before the reform and October 20, 2021–October 2022 after the reform). The dependent variables encompassed the year, claim quantity, dosage, drug cost ratio and health insurance reimbursement amount. ITSA was employed for data analysis. To assess the changes in levels and trends of these indicators before and after the policy implementation, segmented linear multiple regression equations were established, as follows (17):


$$\begin{array}{l} Yt=\beta_{0}+\beta_{1}*\;\mathrm{time}\;+\beta_{2}*\;\mathrm{intervention}\\ \;+\beta_{3}*\;\mathrm{time}:\mathrm{after}:\mathrm{intervention}+\varepsilon \end{array}$$

*Yt* represents the monthly outcome variable observed from October 2020 to October 2022. Time is a continuous variable, denoting the duration in months over the course of the study. The intervention variable serves as an indicator, distinguishing between the pre-intervention period (intervention = 0) and the post-intervention period (intervention = 1) following the policy’s introduction. Additionally, the time after the intervention is represented as a continuous variable, coded as 0 for the month of policy implementation and as continuous numbers for the months preceding and succeeding the policy’s implementation. The regression coefficient *β*_0_ denotes the initial level or intercept at time 0, whereas *β*_1_ signifies the pre-policy release trend or slope of change. Additionally, *β*_2_ represents the immediate level change subsequent to the implementation of the national-negotiated drug policy, and *β*_3_ signifies the post-policy implementation trend or slope of change in the outcome. Consequently, the sum of *β*_1_ and *β*_3_, denoted as *β*_1_ + *β*_3_, represents the true trend in outcomes following the policy release and serves as an indicator of the overall impact of the policy intervention. The random error term ε at moment t remains unexplained within the model.

Segmented linear regression methods were employed to fit equations for multiple linear regression models, with the presence of autocorrelation frequently observed in time series data. The Durbin–Watson test was used to evaluate autocorrelation, where a value of approximately 2 signifies the absence of autocorrelation. In cases where the variables exhibited autocorrelation, the generalized least squares approach (specifically the Prais–Winsten method) was employed to rectify first-order serial correlation errors (18). A significance level of *p* < 0.05 was employed to determine statistical significance. Furthermore, the Dickey–Fuller test was used to evaluate the data’s robustness. All *p*-values obtained from the Dickey–Fuller tests conducted in this study were found to be below the threshold of 0.05.

## Results

### Basic information on drug use in the Xuzhou national negotiation

Over the course of 2 years, the number of individuals using drugs procured through state negotiations in Xuzhou escalated from 3,827 in October 2020 to 11,766 in October 2022, signifying a substantial increase of 7,939. Concurrently, the mean total medical expenses incurred by insured individuals exhibited a decline from 8217.22 in October 2020 to 5418.50 in October 2022. Similarly, the average medical insurance payment cost per patient also experienced a reduction from 8217.22 in October 2020 to 5418.50 in October 2022. The average medical expenses per patient in employees’ medical insurance decreased from 8193.20 to 4826.13 yuan. Similarly, the average medical expenses per patient in medical insurance for urban and rural residents decreased from 8231.37 to 5779.73 yuan. The average medical insurance payment cost per patient experienced a decrease from 3816.76 to 3207.68 yuan. Furthermore, the average medical insurance payment cost per patient for employees participating in medical insurance decreased from 3509.76 to 3315.06, whereas the average medical insurance payment cost per patient for urban and rural residents participating in medical insurance dropped from 3997.67 to 3142.20. The average reimbursement rate for drugs obtained through state-negotiated medical insurance for both urban and rural residents in Xuzhou exhibited an increment from 50 to 60%. Additionally, the average reimbursement rate for medical insurance participants who are employees experienced a rise from 55 to 70% (Supplementary material).

### Overall changes of drugs negotiated by the state in Xuzhou City

**Figure 1:**
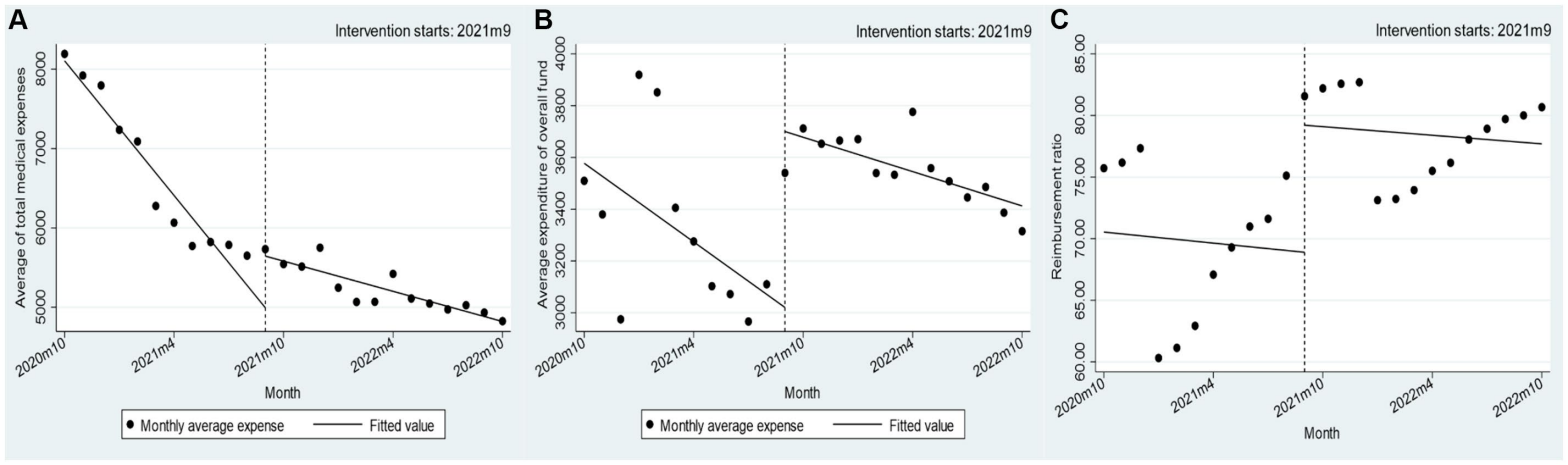
Changes in the cost of negotiated drugs for insured people in Xuzhou, 2020–2022. **(A–C)** Nodes: monthly average expense; Lines: fitted value.

Prior to the implementation of the policy alteration, the average medical expenses per patient of drugs negotiated by the state in Xuzhou exhibited a notable decline, with a monthly reduction of 262.48 yuan (*p* < 0.001), suggesting statistical significance. Despite a subsequent increase of 744.02 yuan in *per capita* expenses following the policy change, a continued downwards trend of 62.42 yuan per month was observed subsequent to the reform (Figure 1). Prior to the implementation of the policy change, there was a statistically significant decrease of $83.11 per month (*p* < 0.001) in the average medical insurance payment cost per patient. Subsequently, following the policy change, there was an immediate increase of $891.65 in the average medical insurance payment cost per patient, followed by a subsequent decrease of $44.13. The alteration in the average reimbursement rate of drugs negotiated by the state did not exhibit a significant change before and after the policy implementation, with only a 7.761% instantaneous increase. Further information can be found in Table 1.

**Table 1:** ITSA results of the policy effect on average medical expenses per patient.

Variables	Coefficient	SE	*t*	*p* value	95% CI	DW
**Entire population**						
β1: baseline slope	−262.48	32.12	−8.17	<0.001	(−329.28, −195.68)	1.72
β2: level change after policy	744.02	261.86	2.84	0.01	(199.46, 1288.59)	
β3: slope change after policy	200.03	33.95	5.89	<0.001	(129.42, 270.64)	
β0: baseline level	8232.32	182.19	45.19	<0.001	(7853.43, 8611.2)	
**Resident**						
β1: baseline slope	−249.43	38.58	−6.46	<0.001	(−329.66, −169.19)	1.81
β2: level change after policy	814.68	303.32	2.69	0.014	(183.89, 1445.47)	
β3: slope change after policy	185.98	41.37	4.5	<0.001	(99.94, 272.02)	
β0: baseline level	8306.2	235.95	35.2	<0.001	(7815.51, 8796.89)	
**Employee**						
β1: baseline slope	−282.88	24.01	−11.78	<0.001	(−332.81, −232.95)	1.47
β2: level change after policy	650.68	212.51	3.06	0.006	(208.74, 1092.62)	
β3: slope change after policy	219.47	24.94	8.8	<0.001	(167.61, 271.32)	
β0: baseline level	8106.69	107.8	75.2	<0.001	(7882.51, 8330.88)	

### Changes in the cost of drugs negotiated by the state for medical insurance participants among urban and rural residents in Xuzhou

**Figure 2:**
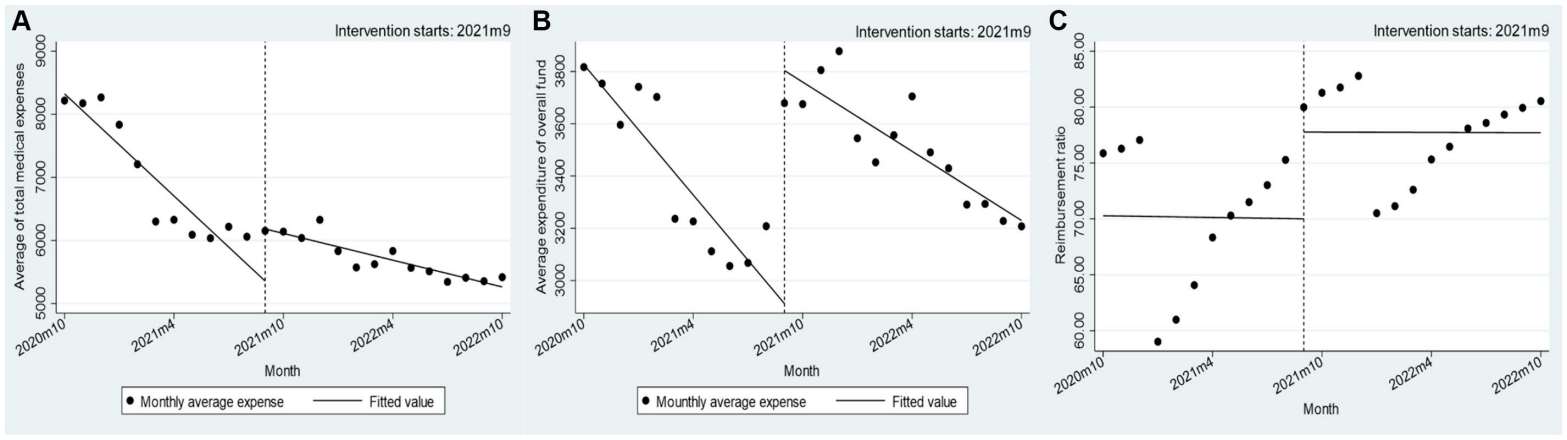
Changes in negotiated drug costs for residents’ health insurance participants, 2020–2022. **(A–C)** Nodes: monthly average expense; Lines: fitted value.

Prior to the implementation of the policy alteration, the mean monthly reduction in total medical expenses for individuals enrolled in urban and rural residents’ medical insurance was 249.43 yuan. Subsequent to the policy change, this reduction decreased to 63.45 yuan per month. The statistical analysis revealed a *p*-value of <0.001, suggesting a significant difference. The implementation of the policy resulted in an immediate increase of 1018.9 yuan in the average medical insurance payment cost per patient for urban and rural residents’ medical insurance participants, followed by a monthly decrease of 57.56 yuan. However, the change in the average annual rate of return for these participants before and after the policy implementation is not statistically significant, as shown in Table 2 (Figure 2).

**Table 2:** ITSA results of the average medical insurance payment cost per patient.

Variables	Coefficient	SE	*t*	*p* value	95% CI	DW
**Entire population**						
β1: baseline slope	−83.11	12.19	−6.82	<0.001	(−108.45, −57.76)	1.53
β2: level change after policy	891.65	117.06	7.62	<0.001	(648.21, 1135.08)	
β3: slope change after policy	38.98	13.8	2.83	0.01	(10.29, 67.78)	
β0: baseline level	3826.13	60.46	63.28	<0.001	(3700.39, 3951.87)	
**Resident**						
β1: baseline slope	−102.359	18.4154	−5.56	<0.001	(−140.66, −64.06)	1.66
β2: level change after policy	1018.9	171.188	5.95	<0.001	(662.90, 1374.91)	
β3: slope change after policy	44.801	20.4327	2.19	0.04	(2.31, 87.29)	
β0: baseline level	3974.607	84.749	46.9	<0.001	(3798.36, 4150.85)	
**Employee**						
β1: baseline slope	−50.63	21.04	−2.41	0.03	(−94.39, −6.88)	1.69
β2: level change after policy	679.56	98.94	6.87	<0.001	(473.80, 885.32)	
β3: slope change after policy	28.52	21.93	1.30	0.21	(−17.10, 74.13)	
β0: baseline level	3577.61	181.4	19.72	<0.001	(3200.37, 3954.85)	

### Changes in the cost of drugs negotiated by the state for employees’ medical insurance participants in Xuzhou

**Figure 3:**
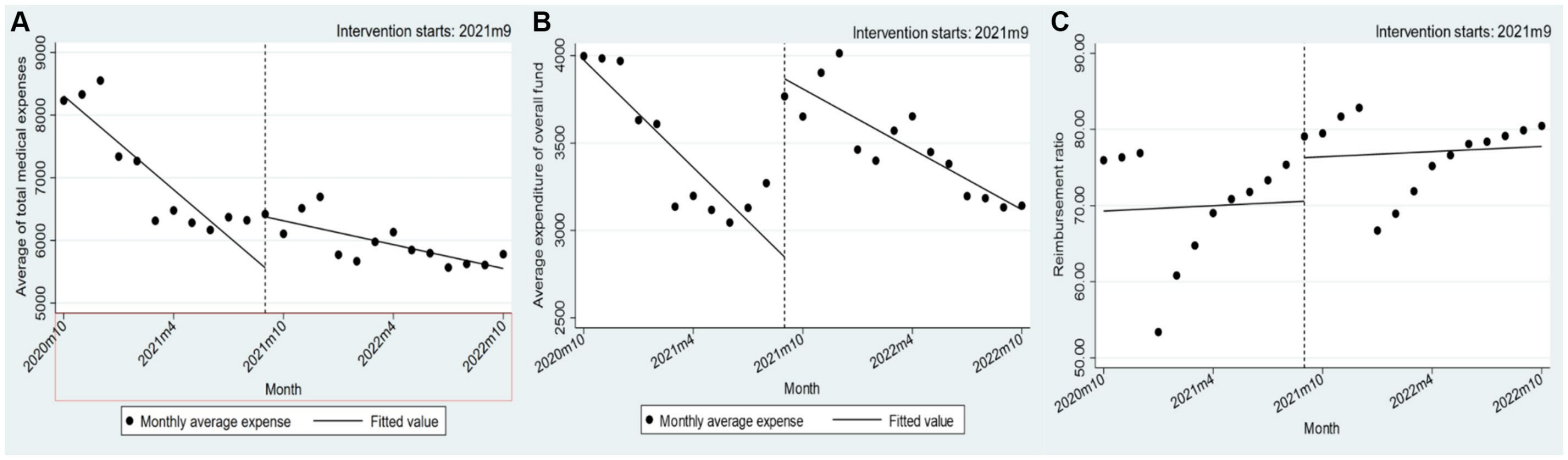
Changes in the cost of drugs negotiated by the state for employees’ medical insurance participants in 2020–2022. **(A–C)** Nodes: monthly average expense; Lines: fitted value.

Prior to the implementation of the policy alteration, there was a notable reduction of 282.88 yuan per month in the average medical expenses per patient incurred by employees enrolled in medical insurance. However, immediately following the policy change, there was a significant increase of 650.68 yuan per month, which subsequently decreased by 63.41 yuan per month. Similarly, the average medical insurance payment cost per patient for employees participating in medical insurance experienced a decrease of 50.63 yuan per month prior to the policy change, followed by an instantaneous increase of 679.56 yuan per month after the policy change. Subsequently, there was a decrease of 22.11 yuan per month after the improvement in treatment. For further details, please refer to Table 3. The average annual rate of return for employees enrolled in medical insurance experienced a sudden increase of 10.32%. However, there was no statistically significant difference observed before and after the policy implementation, as shown in Figure 3.

**Table 3:** ITSA results for the effect of policies on actual reimbursement ratios.

Variables	Coefficient	SE	*t*	*p* value	95% CI	DW
**Entire population**						
β1: baseline slope	−0.025	0.562	−0.04	0.97	(−1.19, 1.15)	1.87
β2: level change after policy	7.761	3.677	2.11	0.05	(0.12, 15.41)	
β3: slope change after policy	0.02	0.606	0.03	0.97	(−1.24, 1.28)	
β0: baseline level	70.281	4.207	16.70	0.00	(61.53, 79.03)	
**Resident**						
β1: baseline slope	0.12	0.65	0.18	0.86	(−1.23, 1.47)	1.81
β2: level change after policy	5.73	4.03	1.42	0.17	(−2.65, 14.10)	
β3: slope change after policy	0.01	0.7	−0.01	1.00	(−1.46, 1.45)	
β0: baseline level	69.28	5.1	13.59	0.00	(58.68, 79.89)	
**Employee**						
β1: baseline slope	−0.15	0.56	−0.27	0.79	(−1.31, 1.01)	1.70
β2: level change after policy	10.32	3.65	2.83	0.01	(2.73, 17.90)	
β3: slope change after policy	0.03	0.6	0.06	0.96	(−1.20, 1.27)	
β0: baseline level	70.54	4.02	17.56	0.00	(62.19, 78.89)	

## Discussion

### Summary of results

This section presents a comprehensive overview of the alterations observed in the mean aggregate medical expenditures, average medical insurance payment cost per patient and mean annual reimbursement rate among insured individuals residing in Xuzhou City subsequent to the enactment of the national drug negotiation policy. The research studies presented here provide evidence of the notable efficacy of this policy in mitigating medical expenditures and facilitating expense coordination, thereby yielding a discernible direct intervention impact. Since the inception of the policy, a consistent decrease in both the mean aggregate medical expenses and the average medical insurance payment cost per patient has been observed, commencing in the initial month. Furthermore, the enduring effectiveness of the policy intervention is supported by the consistent decline observed within the initial year of implementation. This discovery provides substantial evidence for the positive influence of national drug policy negotiations in mitigating the financial strain on patients and bestowing significant advantages upon a substantial population.

### National negotiation of drugs to reduce costs has an effect

Xuzhou City’s reform of the NDPN policy resulted in a significant decrease. The reduction amounted to 63.45 yuan per month (*p* < 0.001) for urban and rural residents and 63.41 yuan per month (*p* < 0.001) for employees. This indicates a consistent downwards trend in the average medical expenses per patient for both groups, with a comparable magnitude of reduction. This phenomenon can be attributed to the state’s drug policy, which primarily focuses on clinically necessary, effective, yet costly drugs, irrespective of the type of insurance. Consequently, the resulting reduction in medical expenses is comparable. In terms of the average medical insurance payment cost per patient, urban and rural residents’ medical insurance participants experienced a decrease of 57.56 yuan (*p* < 0.04), whereas employees’ medical insurance participants witnessed a decrease of 22.11 yuan in their average medical insurance payment cost per patient (*p* = 0.21). The disparity in the reduction in overall payment is evident, potentially attributed to variations in the actual reimbursement rate across different medical insurance categories. Notably, the actual reimbursement rate for urban and rural residents in Xuzhou witnessed an increase from 50% in 2020 to 60% in 2021, whereas the actual reimbursement rate for employees rose from 55% in 2020 to 70% in 2021. As reimbursement rates escalate, medical expenses decrease, resulting in a considerable and substantial decline in the overall payment cost for employees’ medical insurance compared to that of residents’ medical insurance.

One intriguing observation pertains to the abrupt surge in average medical expenses per patient and monthly expenses at the onset of the year. This phenomenon can be attributed to the settlement of critical illness insurance in Xuzhou, where patients who incur higher fees receive supplementary compensation. Consequently, medical expenses toward the end of the year surpass those at the commencement of the subsequent year, thereby elucidating the immediate rise in expenses following the intervention (19).

### Comparison with other studies

Other countries (like Germany and Switzerland) have also adopted a negotiated approach to reduce drug prices and ease the burden on patients. For example, Germany (20) and Switzerland (21) have resolved the problem of high prices of anti-cancer drugs through drug price negotiations. Although the United States has not conducted any drug price negotiation, its drug prices are on an upward trend. After the implementation of price negotiation in Germany, the price of drugs in the United States is higher than that in Germany, and the price gap between the two countries has widened (22).

Previous studies have underscored the impact of drug policy reform on the affordability and accessibility of pharmaceuticals and its potential to alleviate economic burdens for insurance plan beneficiaries, aligning with our own findings (23). Following the implementation of the national drug negotiation policy in Xuzhou, patients have experienced a reduction in economic pressures, although with variations across different medical insurance categories. A national survey on medicines conducted in 11 Chinese provinces and cities found that the financial burden of employee health insurance is relatively low compared to urban and rural residents’ health insurance participants, but the gap is narrowing (24). The financial burden associated with medical insurance for employees is comparatively lower than that for participants in urban and rural residents’ medical insurance, although with a narrower disparity. This discrepancy may stem from the distinct resource allocations in the design of basic medical insurance systems for urban workers and urban and rural residents. Urban and rural residents exhibit lower levels of and economic strength than urban workers.

However, the singular focus on minimizing medical costs may inadvertently result in patients incurring higher expenses for examinations, materials and nursing care. The findings of a number of scholars suggest that the alteration of drug policies can potentially influence the practices of healthcare providers in China, consequently leading to elevated costs for medications, medical supplies and out-of-pocket expenditures subsequent to the implementation of drug reforms (10, 25). Certain hospitals and doctors induce patients’ demand for medical care, which in turn increases patients’ use of medical services to protect the hospital’s income (26). It is recommended that medical insurance benefits be established in a reasonable manner to mitigate disparities in treatment among patients with varying insurance types. Simultaneously, it is essential to foster the growth of local supplementary medical insurance, investigate system design to enhance the cost-effectiveness of medical insurance funds, enhance the establishment of a multi-tiered medical insurance system and augment security efficiency.

### Advantages of the present study

This study employs a comprehensive approach by selecting data from a 24-month period prior to and following the implementation of Xuzhou City’s 2021 reform of the national drug policy negotiation. The objective of this study is to thoroughly assess the long-term effects of this policy. The analysis focuses on comparing two distinct categories of basic medical insurance in China, specifically employee medical insurance and urban and rural residents’ medical insurance.

### Limitations of the present study

However, this study is subject to certain limitations. First, the observation of national drug negotiation costs prior to 2020 was hindered by the limited availability of data within the specified time frame. Second, the significant variation in the implementation methods of national drug policy negotiation across provinces precludes the identification of a suitable control group to investigate factors other than policy implementation that contribute to changes in drug usage. Third, the scope of this study is confined to Xuzhou City, Jiangsu Province, thereby limiting the generalizability of the findings to regions with similar economic characteristics. This study employs the average medical expenses per patient, the average medical insurance payment cost per patient and the actual reimbursement ratio as indicators to assess the influence of national-negotiated drugs on the economic burden of residents’ diseases. However, notably, these indicators exhibit strong consistency, suggesting the presence of internal mixed factors. Furthermore, it is crucial to recognize that a reduction in medical expenses does not automatically translate to an enhancement of fairness. Therefore, in future reforms, greater attention should be directed toward achieving equalization of financing and welfare to thoroughly investigate the implementation effect of the national drug negotiation policy.

## Conclusion

According to the results of China’s National Drug Negotiation in 2023, 143 off-catalog drugs participated in the negotiation, of which 121 drugs were successfully negotiated, and drug prices were reduced by an average of 61.7%. Xuzhou City, through the implementation of the NDPN policy, so that some drugs zero price difference into the pharmacy, not only opened up the sales channels of prescription drugs, so that patients to buy drugs more convenient, but also to reduce the burden on patients. This study demonstrates that the implementation of the Xuzhou City’s 2021 reform of the NDPN policy effectively decreases the average medical expenses per patient, partially alleviating the healthcare burden on residents. Furthermore, this study ensures the equitable functioning of the medical insurance fund and potentially fosters the sustainable development of said fund. Consequently, the findings of this study offer valuable insights for other regions and countries seeking to mitigate citizens’ healthcare expenses.

## Author contributions

ZQ: Data curation, Formal analysis, Supervision, Writing – original draft, Writing – review & editing. MH: Data curation, Formal analysis, Writing – original draft, Writing – review & editing. HS: Data curation, Writing – original draft, Writing – review & editing. SL: Supervision, Writing – original draft, Writing – review & editing. SX: Data curation, Visualization, Writing – original draft, Writing – review & editing. LC: Data curation, Writing – original draft, Writing – review & editing.
