# Supplementary material for: The influence of the national drug price negotiation policy reform on the medical expenses of patients in Xuzhou City: an interrupted time series analysis
Source: Front Public Health. 2024 Jun 5;12:1381786. doi: 10.3389/fpubh.2024.1381786 (PMC11188421; doi:10.3389/fpubh.2024.1381786)
Supplement: Supplementary file 1 [file Table_1.docx]

Basic information on the use of single line payment drugs in Xuzhou

| Date | person-time | | | Average total medical expenses per patient | | | Average of pooled fund expenditures per patient | | | Actual reimbursement ratio | | |
| --- | --- | --- | --- | --- | --- | --- | --- | --- | --- | --- | --- | --- |
|  | Totality | Resident | Employee | Totality | Resident | Employee | Totality | Resident | Employee | Totality | Resident | Employee |
| 20-Oct | 3827 | 2408 | 1419 | 8217.22 | 8231.37 | 8193.20 | 3816.76 | 3997.67 | 3509.76 | 75.87 | 75.95 | 75.72 |
| 20-Nov | 4373 | 2704 | 1669 | 8173.71 | 8329.59 | 7921.16 | 3753.66 | 3984.29 | 3380.02 | 76.28 | 76.34 | 76.18 |
| 20-Dec | 5466 | 3414 | 2052 | 8267.49 | 8550.81 | 7796.11 | 3596.01 | 3969.28 | 2974.98 | 77.05 | 76.90 | 77.34 |
| 21-Jan | 4679 | 2894 | 1785 | 7299.49 | 7338.37 | 7236.44 | 3741.16 | 3631.18 | 3919.46 | 59.02 | 53.40 | 60.32 |
| 21-Feb | 4163 | 2566 | 1597 | 7198.76 | 7266.58 | 7089.79 | 3702.36 | 3609.50 | 3851.58 | 60.99 | 60.83 | 61.14 |
| 21-Mar | 6226 | 3914 | 2312 | 6300.04 | 6313.28 | 6277.63 | 3236.53 | 3136.54 | 3405.79 | 64.08 | 64.76 | 62.93 |
| 21-Apr | 6690 | 4242 | 2448 | 6327.78 | 6478.46 | 6066.67 | 3226.35 | 3197.90 | 3275.64 | 68.34 | 69.02 | 67.08 |
| 21-May | 6487 | 4045 | 2442 | 6090.62 | 6282.21 | 5773.25 | 3112.15 | 3117.81 | 3102.77 | 70.30 | 70.86 | 69.29 |
| 21-Jun | 7105 | 4409 | 2696 | 6036.18 | 6166.58 | 5822.91 | 3055.80 | 3045.73 | 3072.27 | 71.50 | 71.80 | 70.99 |
| 21-Jul | 7207 | 4460 | 2747 | 6148.43 | 6371.04 | 5787.01 | 3067.62 | 3130.02 | 2966.32 | 73.02 | 73.33 | 71.61 |
| 21-Aug | 7495 | 4566 | 2929 | 6059.44 | 6321.47 | 5650.95 | 3208.19 | 3270.99 | 3110.29 | 75.28 | 75.37 | 75.12 |
| 21-Sep | 8355 | 5105 | 3250 | 6152.92 | 6420.08 | 5733.27 | 3678.97 | 3767.20 | 3540.39 | 79.98 | 79.08 | 81.57 |
| 21-Oct | 6959 | 4260 | 2699 | 5888.37 | 6105.92 | 5545.01 | 3675.21 | 3651.79 | 3712.17 | 81.28 | 79.49 | 82.21 |
| 21-Nov | 8602 | 5255 | 3347 | 6124.11 | 6512.93 | 5513.64 | 3805.35 | 3902.37 | 3653.03 | 81.75 | 81.69 | 82.58 |
| 21-Dec | 9614 | 5868 | 3746 | 6327.46 | 6695.18 | 5751.42 | 3877.77 | 4013.44 | 3665.25 | 82.79 | 82.84 | 82.70 |
| 22-Jan | 9271 | 5625 | 3646 | 5565.46 | 5771.70 | 5247.28 | 3544.56 | 3462.84 | 3670.63 | 70.51 | 66.72 | 73.13 |
| 22-Feb | 8161 | 5079 | 3082 | 5441.46 | 5668.26 | 5067.72 | 3452.45 | 3399.48 | 3539.74 | 71.14 | 68.93 | 73.22 |
| 22-Mar | 10553 | 6458 | 4095 | 5624.67 | 5976.79 | 5069.37 | 3556.34 | 3571.11 | 3533.06 | 72.61 | 71.89 | 73.94 |
| 22-Apr | 8276 | 4802 | 3474 | 5833.88 | 6132.25 | 5421.45 | 3704.72 | 3653.07 | 3776.11 | 75.32 | 75.20 | 75.51 |
| 22-May | 11142 | 6907 | 4235 | 5567.37 | 5847.13 | 5111.09 | 3490.62 | 3448.92 | 3558.62 | 76.46 | 76.62 | 76.16 |
| 22-Jun | 11602 | 7188 | 4414 | 5511.60 | 5796.46 | 5047.72 | 3429.65 | 3381.57 | 3507.96 | 78.08 | 78.10 | 78.05 |
| 22-Jul | 11771 | 7330 | 4441 | 5343.45 | 5567.71 | 4973.31 | 3290.61 | 3196.71 | 3445.59 | 78.58 | 78.39 | 78.93 |
| 22-Aug | 13223 | 8462 | 4761 | 5410.01 | 5625.46 | 5027.10 | 3293.26 | 3184.79 | 3486.06 | 79.33 | 79.14 | 79.71 |
| 22-Sep | 13431 | 8384 | 5047 | 5354.73 | 5607.94 | 4934.10 | 3228.03 | 3132.44 | 3386.83 | 79.93 | 79.89 | 80.01 |
| 22-Oct | 11766 | 7309 | 4457 | 5418.50 | 5779.73 | 4826.13 | 3207.68 | 3142.20 | 3315.06 | 80.54 | 80.46 | 80.68 |
